# Personality to Prescription Drug Misuse in Adolescents: Testing Affect Regulation, Psychological Dysregulation, and Deviance Proneness Pathways

**DOI:** 10.3389/fpsyt.2021.640766

**Published:** 2021-04-27

**Authors:** Sherry H. Stewart, Annie Chinneck, Kara Thompson, Mohammad H. Afzali, Raquel Nogueira-Arjona, Ioan T. Mahu, Patricia J. Conrod

**Affiliations:** ^1^Department of Psychiatry, Dalhousie University, Halifax, NS, Canada; ^2^Department of Psychology & Neuroscience, Dalhousie University, Halifax, NS, Canada; ^3^Department of Psychology, St. Francis Xavier University, Antigonish, NS, Canada; ^4^Department of Psychiatry, Université de Montréal, Montréal, PQ, Canada

**Keywords:** adolescents, personality risk, prescription drug misuse, anxiety sensitivity, hopelessness, sensation seeking, impulsivity, mental health symptoms

## Abstract

**Background:** Fifteen to 25-year-olds are the age group most likely to misuse prescription drugs. Few studies have tested theory-driven models of adolescent risk for prescription drug misuse. Moreover, rarely are distinct pathways to different forms of prescription drug misuse considered.

**Methods:** We tested mediational paths from personality to mental health symptoms to prescription drug misuse, informed by etiological models of addiction. We specified pathways from particular personality traits to unique forms of prescription drug misuse via specific mental health symptoms. We used semi-longitudinal data collected across two waves of the Co-Venture Trial. Our sample included students from 31 Canadian high schools tested in Grade 9 (*n* = 3,024) and again in Grade 10 (*n* = 2,869; 95% retention). Personality (hopelessness, anxiety sensitivity, impulsivity, sensation seeking) was assessed in Grade 9. Mental health symptoms (depression, anxiety, ADHD, conduct disorder) and prescription drug misuse (opioids, sedatives/tranquilizers, stimulants) were assessed at both time points.

**Results:** Consistent with the negative affect regulation model, hopelessness was specifically associated with opioid misuse via depressive symptoms, and anxiety sensitivity was specifically associated with sedative/tranquilizer misuse via anxiety symptoms. Consistent with positive affect regulation, sensation seeking was directly associated with stimulant misuse. Consistent with the psychological dysregulation model, impulsivity was associated with stimulant misuse via ADHD symptoms. And consistent with the deviance proneness model, impulsivity was also associated with unconstrained (i.e., all three forms of) prescription drug misuse via conduct disorder symptoms.

**Conclusions:** Screening for adolescents high in hopelessness, anxiety sensitivity, sensation seeking, or impulsivity and providing them with personality-matched cognitive-behavioral interventions may be helpful in preventing or mitigating prescription drug misuse. Our results point to the specific mental health symptoms that are important to target in each of these personality-matched interventions.

## Introduction

The National Survey on Drug Use and Mental Health defines prescription drug (PD) misuse as use of PDs “in any way that a doctor did not direct you to use them” including (a) use without a prescription of one's own; (b) use in greater amounts, for longer, or more often than prescribed; or (c) use in any other way that was not prescribed by a physician (1). Many young people consider PDs to be less harmful than illicit drugs (2). Due to their potency, potential for addiction, and overdose potential, however, PD misuse can be injurious or even fatal (3).

Of any age group, 15–25-year-olds are the most likely to misuse PDs (1). After cannabis, PDs are the drugs most commonly misused by North American adolescents (1, 4). One study showed that among adolescents aged 12–17, 5% reported past year PD misuse (5). PDs are readily accessible to adolescents through legitimate medical prescriptions (6), diversion (7, 8), and online pharmacies (9, 10). These trends are concerning for several reasons. First, prescription opiate misuse increases risk for serious injury (11), respiratory depression, and death (12). Moreover, the prevalence of adolescent misuse of sedatives/tranquilizers, including novel designer benzodiazepines, is significantly increasing (13–15), co-use with opioids is common (16), and sedative/tranquilizer-related deaths increased by 137% from 2007 to 2016 (17). Stimulant misuse is associated with adverse short-term (e.g., headaches, sleep problems, academic difficulties) and long-term effects [e.g., decreased likelihood of college graduation; (18)]. Adolescent-onset PD misuse is linked with elevated substance use disorder rates in adulthood (18, 19).

While several risk and protective factors for adolescent PD misuse have been identified [see review by (20)], few studies have tested theoretical models of adolescent risk for PD misuse (21). And although the predictors of PD misuse may vary considerably by drug class (22), little work has examined unique pathways to specific forms of PD misuse. One potential risk factor that may help fill both these identified gaps is personality: specific traits may present risk for particular classes of PD misuse via unique theory-informed pathways.

### Personality as a Risk Factor

Personality is a robust predictor of addictive behavior [e.g., (23)]. Internalizing and externalizing traits have been reliably associated with an increased susceptibility for alcohol and illicit substance misuse in adolescence (24). Pihl and Peterson (25) developed a model that delineates four such traits. The first two traits in this model are internalizing. Hopelessness (HOP) involves the trait-like tendency to expect aversive events but not desirable ones (26, 27). Anxiety sensitivity (AS) involves the fear of anxiety-related sensations, due to an unrealistic expectation that such sensations will have catastrophic consequences (28). In adolescents, both HOP and AS are associated with coping motives for substance use (29). Young people high in these traits tend to preferentially misuse depressant drugs (30, 31). In adults, HOP uniquely predicts opioid dependence and AS uniquely predicts anxiolytic dependence (30, 32). The specificity of these paths has yet to be tested in adolescents.

The remaining two traits in Pihl and Peterson's (25) model are externalizing. Impulsivity (IMP), or impulsiveness, is the tendency to act without sufficient forethought (33). IMP has been associated with a pattern of polysubstance use (34, 35). Deficits in response inhibition make high IMP teens more susceptible to early experimentation and to later compulsive substance use (36). Sensation seeking (SS), or novelty seeking (37), involves the desire for novel and intense stimulation (38). High SS substance users are sensitive to the rewarding properties of drugs (39) and tend to specifically misuse stimulants (40) to study, stay awake/alert, “get high,” “party,” and experiment (41).

Traits from Pihl and Peterson's (25) four-factor personality vulnerability model have proven useful in predicting adolescent alcohol (42) and illicit drug use (43, 44), emerging adult PD use (31, 45), and adult PD use (30). This model has yet to be applied to adolescent PD misuse.

### Etiological Models of Addiction

Theoretically, these four traits exert their influence on substance use via negative and positive affect regulation, deviance proneness, and/or psychological dysregulation processes (39). The models most relevant to linking HOP, AS, SS, and IMP with PD misuse are described below (see also Table 1). These theoretical models have informed the mediators in the hypothesized paths from personality to PD misuse.

**Table 1:** Summary of theories and hypotheses.

**Personality trait**	**Relevant etiological model**	**Derived hypotheses**
HOP, AS	Negative affect regulation	*H1*: HOP → depressive symptoms → opioid misuse *H2*: AS → anxiety symptoms → sedative/tranquilizer misuse
SS	Positive affect regulation	*H3*: SS → stimulant misuse
IMP	Deviance proneness	*H4*: IMP → CD symptoms → opioid misuse IMP → CD symptoms → sedative/tranquilizer misuse IMP → CD symptoms → stimulant misuse
IMP	Psychological dysregulation	*H5*: IMP → ADHD symptoms → stimulant misuse

#### Affect Regulation Models

Affect regulation models theorize that drugs are taken to regulate emotions—either for negative reinforcement (i.e., a drug's ability to relieve negative affect) or positive reinforcement (i.e., a drug's hedonic effects) (31). Negative affect regulation involves PD use to avoid or control negative emotional states whereas positive affect regulation involves PD use to increase positive emotional states. This dichotomy is in keeping with McCabe et al.'s (46) work on PD misuse motives, which suggests that PDs are misused for self-medication (negative affect regulation) or recreation (positive affect regulation).

##### Negative Affect Regulation

Individuals high in HOP or AS are theoretically most prone to PD misuse for negative affect regulation (29). First, those high in HOP are thought to misuse opioids to control or avoid symptoms of depression. High HOP adults preferentially misuse opioids over other substances (30–32). HOP also predicts adolescent depression (47), and depression increases risk of PD misuse (21). The negative affect regulation model suggests that depressive symptoms should mediate HOP's specific effect on opioid misuse.

Those high in AS are also theoretically prone to PD misuse for negative affect regulation but through a distinct pathway. Specifically, they are thought to misuse sedatives/tranquilizers to control or avoid anxiety symptoms. High AS adults preferentially misuse anxiolytics over other substances (30, 31). AS incrementally predicts anxiety disorder symptoms in children and adolescents (48, 49) and anxiety disorders are associated with increased risk for sedative/tranquilizer misuse (50). In sum, the negative affect regulation model supports two distinct and specific pathways: HOP to opioid misuse via depressive symptoms vs. AS to sedative/tranquilizer misuse via anxiety symptoms.

##### Positive Affect Regulation

Stimulants activate mesolimbic dopamine activity and increase positive mood (51). High SS individuals are theoretically most prone to stimulant misuse for positive affect regulation. SS is robustly related to sensitivity to drug reward (39) and to enhancement motivated substance use (31). High SS individuals preferentially misuse stimulants (32, 40). The positive affect regulation model suggests this is because SS underlies sensitivity to stimulant reinforcement (52). The positive affect regulation model suggests a direct pathway from SS to stimulant misuse that is not mediated through mental health symptoms.

#### Deviance Proneness Model

Another model relevant to understanding PD misuse is the deviance proneness model (53). High IMP individuals are thought to be prone to a broad, unconstrained pattern of PD misuse (opioid, sedative/tranquilizer, and stimulant), occurring amidst other “deviant” or antisocial behaviors. IMP is associated with comorbid addictive and antisocial behaviors (54). IMP in elementary school students is concurrently and prospectively associated with conduct problems (55). Conduct disorder (CD) symptom severity is associated with greater substance involvement (56), including unconstrained PD misuse (57), in adolescence. The deviance proneness model suggests that CD symptoms mediate IMP's effect on unconstrained PD misuse (i.e., all three types of PD misuse).

#### Psychological Dysregulation Model

The psychological dysregulation model is an alternative model for explaining the specific link of IMP to stimulant misuse. Individuals high in IMP are most prone to PD misuse resulting from an adverse environment triggering a heritable tendency toward psychological dysregulation (58). ADHD is an externalizing disorder characterized by high IMP (59). Individuals with ADHD (60) or high IMP levels (24) are more likely to misuse stimulants. While only 4% of 10–18-year-olds endorse past-month stimulant misuse (61), 14% of 4–17-year-olds with ADHD endorse past-2-week stimulant misuse (62). IMP's effect on stimulant misuse may be attributable, at least in part, to an inability to inhibit pre-potent responses (63). ADHD symptoms are associated with stimulant misuse even after controlling for prescribed use (64). The psychological dysregulation model suggests that symptoms of ADHD mediate IMP's specific effect on stimulant misuse.

### Objectives

Nargiso et al. (20) reviewed 50 articles on adolescent PD misuse and identified the following limitations. First, most studies were cross-sectional. Second, non-demographic risk factors (e.g., personality, mental health symptoms) were understudied. Third, there was a lack of specificity regarding predictors of misuse across PD classes. The present study sought to address these limitations by examining predictors of different forms of PD misuse (i.e., opioid, sedative/tranquilizer, stimulant) in a large sample of Canadian adolescents, tested prospectively in Grades 9 and 10 through a “semi-longitudinal design.” In this design, one part is longitudinal (i.e., tests of personality to mental health symptoms and personality to PD misuse) and the other part is cross-sectional (i.e., tests of mental health symptoms to PD misuse). We used a broad definition of PD misuse in the present study, involving use of a PD in any way not directed by a physician (1).

See Table 1 for a summary of our hypotheses. Based on the theories described above, we hypothesized that: in keeping with the negative affect regulation model, (*H1*) Grade 9 HOP would specifically predict Grade 10 opioid misuse via Grade 10 depressive symptoms, and *(H2)* Grade 9 AS would specifically predict Grade 10 sedative/tranquilizer misuse via Grade 10 anxiety symptoms; in keeping with the positive affect regulation model, *(H3)* Grade 9 SS would directly predict Grad 10 stimulant misuse; in keeping with the deviance proneness model, *(H4)* Grade 9 IMP would predict Grade 10 opioid misuse, sedative tranquilizer misuse, and stimulant use, all via Grade 10 CD symptoms; and in keeping with the psychological dysregulation model, *(H5)* Grade 9 IMP would also predict Grade 10 stimulant misuse via Grade 10 ADHD symptoms.

## Methods

The present study's data was archival. It was collected as part of the Co-Venture Trial (65) examining the longer-term efficacy of personality-targeted substance misuse prevention. Assenting students from 31 high schools (public and private; English and French) in Montreal, Canada participated. Data was collected annually (during the fall and spring terms) beginning in September 2012. A web-based platform (Delosis Ltd., London, U.K.) was used to survey students during regular class times. At baseline, students were in Grade 7. The present study used data collected prospectively in Grade 9 (September 2014-May 2015) and Grade 10 (September 2015-May 2016). Risk increases as adolescents transition from middle to high school (66). In Canada, high school normally runs from Grades 9-12 (67). We therefore excluded Grade 7-8 (i.e., middle school) data. Ethical approval was granted by Sainte-Justine Hospital's Research Ethics Board (approval number = 2012-396, 3427) and by each administrative school board.

### Participants

Sample sizes were *n* = 3,024 in Grade 9 and *n* = 2,869 of these same students in Grade 10 (5% attrition). See Table 2 for sample characteristics.

**Table 2:** Frequencies and descriptive statistics.

	**Grade 9**				**Grade 10**			
	***n***	**%**	***M (SD)***	**α**	***N***	**%**	***M (SD)***	**α**
Age	3,024		14.79 (0.47)		2,869		15.83 (0.42)	
**Gender**								
Male	1,463	50.7			1,374	50.1		
Female	1.425	49.3			1,371	49.9		
**Ethnicity**								
Canadian or American	2,535	87.8			2,413	87.9		
European	64	2.2			63	2.3		
African	57	2.0			46	1.7		
Caribbean	28	1.0			26	0.9		
East Asian	81	2.8			82	3.0		
South Asian	17	0.6			17	0.6		
Middle Eastern	21	0.7			21	0.8		
South or Central American	44	1.5			39	1.4		
Other	27	0.9			23	0.8		
Don't know	14	0.5			15	0.5		
Socioeconomic status			5.36 (1.69)				5.37 (1.66)	
Alcohol misuse			0.09 (0.59)	0.79			0.17 (0.79)	0.81
Hopelessness			12.51 (3.92)	0.89			12.73 (3.83)	0.89
Anxiety sensitivity			11.09 (2.95)	0.70			11.02 (2.97)	0.73
Sensation seeking			16.14 (3.63)	0.70			16.37 (3.70)	0.71
Impulsivity			11.66 (2.91)	0.75			11.55 (2.87)	0.75
Depression			5.32 (5.98)	0.90			5.45 (5.93)	0.90
Anxiety			2.81 (4.03)	0.90			2.82 (3.99)	0.89
ADHD			4.12 (2.40)	0.72			4.12 (2.38)	0.74
CD			2.18 (1.64)	0.62			2.09 (1.61)	0.64
Opioids	54	1.8			88	3.1		
Sedatives/tranquilizers	95	3.1			100	3.5		
Stimulants	50	1.7			63	2.2		

*Socioeconomic Status was rated using a 10-point Likert scale (68) with higher scores representing greater wealth. Alcohol Misuse was assessed using the DEP-ADO (69); internal consistency values for the DEP-ADO alcohol misuse scale was calculated only among the more frequent drinkers as others were skipped over these items and assigned a score of zero. Personality was assessed using the SURPS (31). Depression and Anxiety were assessed using the BSI-18 (70). ADHD, Attention-Deficit Hyperactivity Disorder; CD, Conduct Disorder. Both were assessed using the SDQ (71). PD misuse was assessed using the DEP-ADO (69) and scored dichotomously. For short scales with 10 items or less, an alpha of **≥** 0.60 is considered acceptable (72)*.

### Measures

#### Personality

The 23-item Substance Use Risk Profile Scale (SURPS; 30) was used to assess personality as part of the Co-Venture Trial. The SURPS has four subscales: HOP (7 items; “I feel that I'm a failure”), AS (5 items; “It is frightening to feel dizzy or faint”), SS (6 items; “I like doing things that frighten me a little”), and IMP (5 items; “I usually act without stopping to think”). Participants responded using a 5-point Likert scale (1 *strongly disagree* to 5 *strongly agree*). Following reverse scoring of certain negatively keyed items, subscale scores were generated by summing component items. The SURPS was chosen for use in the large-scale Co-Venture survey given its brevity and its strong psychometric properties in both English (43) and French (73). These include acceptable to good internal consistency, factorial validity, convergent and discriminant validity (e.g., with similar personality measures), and concurrent, predictive, and incremental validity in relation to substance use and substance-related problems in youth [e.g., (31, 43, 74)]. In the present sample, the subscales were internally consistent (see Table 2).

#### Internalizing Symptoms

The 18-item Brief Symptom Inventory-18 [BSI-18; (70)] was used to assess depression and anxiety symptoms. It measures past-week psychological distress. In this study, only the Depression (6 items; “feeling blue”) and Anxiety (6 items; “nervousness or shakiness inside”) subscales were used. Participants responded using a 5-point Likert Scale (0 *not at all* to 4 *extremely often*). Subscale scores were generated by summing component items. The BSI-18 has strong psychometric properties in both English (75) and French (76). In our sample, the subscales were internally consistent (see Table 2).

#### Externalizing Symptoms

The 25-item Youth Self-Report Strengths and Difficulties Questionnaire (SDQ; 73) was used to assess ADHD and CD symptoms. It measures symptoms over the past 6-months. In this study, only the Hyperactivity/Inattention (5 items; “restless, cannot sit still for long”) and Conduct Problems (5 items; “often accused of lying or cheating”) subscales were used (77). The remaining subscales were excluded as they pertain instead to prosocial (Prosocial Behavior) and internalizing (Emotional Symptoms, Peer Relationship Problems) behaviors (77). Participants responded using a 3-point Likert Scale (0 *not true* to 2 *certainly true*). Following reverse scoring of certain items, subscale scores were generated by summing component items. The SDQ has strong psychometric properties in both English (78) and French (79). In our sample, the subscales were internally consistent (see Table 2).

####  Prescription Drug Misuse

A modified and validated version of the Detection of Alcohol and Drug Problems in Adolescents (DEP-ADO; 77) assessed lifetime PD misuse for: (1) Opioids: e.g., “Codeine, Demerol, Morphine, Percodan, Methadone, Darvon, Opium, Dilaudid, or Talwin”; (2) sedatives: e.g., “barbiturates, sedatives, downers, or sleeping pills like Seconal and Quaaludes”; (3) tranquilizers: e.g., “Valium, Librium, or Ativan”; and (4) stimulants: e.g., “stimulants, speed, methamphetamine, amphetamine, or Benzedrine.” Participants responded using a 6-point frequency scale (0 *never* to 5 *every day*). To deal with zero-inflation, items were scored dichotomously (i.e., 1 = *had used that PD class*, 0 = *had not*). In keeping with our previous research (45), sedatives and tranquilizers were collapsed into a single category. The DEP-ADO has strong psychometrics and is available in both English (69) and French (80). It was developed for and validated with adolescents aged 14-17 years (i.e., Grades 9–11). It has a strong test-retest reliability (*r* =0.94), acceptable to good internal consistency (Cronbach's alpha =0.61–0.86), and content, convergent, and criterion-related validity (sensitivity =0.84; specificity =0.91) (69).

#### Alcohol Misuse

Alcohol misuse was also assessed using the modified DEP-ADO (69). This scale includes 10 yes/no items that pertain to lifetime issues with: physical health, psychological health, familial relationships, intimate relationships, academics, finances, delinquency, risky behavior, alcohol tolerance, and treatment seeking, attributable to one's alcohol use. This sole focus on alcohol was a change from the original DEP-ADO which asked these items for alcohol and other drugs combined (69). Items were summed to create a 0–10 total score. Only those indicating a frequency of drinking greater than or equal to “weekends or once or twice during the week” on a previous DEP-ADO item were asked these alcohol misuse items; the others were skipped over these items and automatically assigned an alcohol misuse score of 0. In the present sample, the alcohol misuse scale was internally consistent (see Table 2).

### Statistical Analyses

Sample descriptive statistics were first calculated in SPSS 20.0. T-tests and chi square tests were used to compare baseline (Grade 9) characteristics of those retained (*n* = 2,869) vs. lost to follow-up (*n* = 155) in Grade 10. Correlations were specified between the personality, mental health, and PD misuse variables. The hypothesized model was then run in MPlus 7.11 (81). Because our dependent variables were categorical, a robust weighted least squares approach was used [ESTIMATOR = WLSMV; (82)]. Missing data was handled using pairwise deletion such that only those with data at both timepoints were used in hypothesis testing. We controlled for school and for Grade 9 mental health and PD misuse. Our model therefore accounts for new users. We also controlled for age, sex, ethnicity, and socioeconomic status (68), given their known effects on PD misuse (20, 83). Because high-intensity drinking is associated with PD misuse (84), we controlled for alcohol misuse as assessed on the DEP-ADO. These covariates were regressed onto all the outcome variables.

Standard indices were used to assess model fit. RMSEA ≤ 0.05 and CFI/TLI ≥0.95 indicate good fit. RSMEA ≤ 0.08 and CFI/TLI ≥0.90 indicate adequate fit (85). Since chi-square values are often significant when the sample size is large (86), we did not interpret the chi-square as a fit statistic. Instead, we used the χ^2^/*df* ratio where a value <3.0 indicates good fit. Significant effects were detected at a 95% confidence interval. Bootstrapped confidence intervals were used to determine the significance of indirect effects (i.e., significant if the confidence intervals did not cross zero).

## Results

### Sociodemographic Features

On average, students were 14.8 (*SD* = 0.5) years of age in Grade 9. There was a relatively equal split of the sample across gender at both waves. Most students were middle class and of Canadian or American descent (see Table 2).

### Personality

Sample mean scores on the four subscales of the SURPS were relatively consistent with norms on the measure from a previously tested sample of adolescents (31). Scores remained relatively stable from Grade 9 to Grade 10 (see Table 2).

### Mental Health

Sample mean scores on the BSI-18 measure of internalizing mental health symptoms indicated that levels of anxiety and depression symptoms were both relatively low, on average, in our non-clinical sample at baseline (Grade 9), with depression symptom scores somewhat higher than anxiety symptom scores overall. Sample mean scores on the SDQ measure of externalizing mental health symptoms similarly indicated that levels of ADHD and CD symptoms were both relatively low, on average, in our non-clinical sample at baseline (Grade 9), with ADHD symptom scores somewhat higher than CD symptoms scores overall. Scores remained relatively stable on all four measures of mental health symptoms from Grade 9 to Grade 10 (see Table 2).

### Substance Misuse

In Grade 10, lifetime PD misuse rates were: 3% for opioids, 4% for sedatives/tranquilizers, and 2% for stimulants (see Table 2). Rates of misuse of each type of PD rose between Grade 9 and Grade 10 with the sharpest increase observed for opioid misuse. Levels of alcohol misuse also rose between Grade 9 and Grade 10 (see Table 2).

### Comparison of Students Retained vs. Lost to Follow-Up

*T*-tests and chi-square tests suggested that, at baseline (Grade 9), those who were later lost to follow-up (Grade 10) were older, more likely to attend certain schools, and endorsed more personality vulnerability (HOP, SS, IMP), mental health symptoms (depression, CD, ADHD), alcohol misuse, and PD misuse.

### Correlations

Bivariate correlations between study variables are displayed in Table 3. With respect to correlations between Grade 9 personality and Grade 10 mental health symptoms, HOP was most strongly associated with depressive symptoms, AS was most strongly associated with anxiety symptoms, and IMP and SS were most strongly associated with ADHD and CD symptoms (with IMP showing much stronger associations than SS in this regard). With respect to correlations between Grade 10 mental health symptoms and Grade 10 PD misuse, the strongest correlations were between CD symptoms with all three forms of PD misuse, anxiety and depressive symptoms with sedative/tranquilizer misuse, and ADHD symptoms with stimulant misuse. Grade 9 alcohol misuse was significantly associated with all Grade 9 personality factors save AS, with all four measures of Grade 10 mental health symptoms, and with all three forms of PD misuse in Grade 10, underlining the importance of alcohol misuse as a covariate.

**Table 3:** Correlation matrix.

	**1**	**2**	**3**	**4**	**5**	**6**	**7**	**8**	**9**	**10**	**11**	**12**
**Grade 9**												
1. Hopelessness	1.00	**0.27**	−0.03	**0.32**	**0.11**	**0.46**	**0.35**	**0.33**	**0.22**	**0.07**	**0.11**	**0.09**
2. Anxiety sensitivity		1.00	–**0.12**	**0.19**	0.02	**0.23**	**0.33**	**0.14**	**0.05**	−0.03	−0.01	−0.01
3. Sensation seeking			1.00	**0.25**	**0.14**	−0.01	−0.02	**0.12**	**0.16**	**0.13**	**0.11**	**0.11**
4. Impulsivity				1.00	**0.11**	**0.22**	**0.18**	**0.45**	**0.41**	**0.11**	**0.11**	**0.12**
5. Alcohol harms					1.00	**0.08**	**0.06**	**0.08**	**0.13**	**0.12**	**0.17**	**0.20**
**Grade 10**												
6. Depression						1.00	**0.73**	**0.29**	**0.24**	**0.07**	**0.16**	**0.10**
7. Anxiety							1.00	**0.29**	**0.20**	**0.06**	**0.11**	**0.07**
8. ADHD								1.00	**0.40**	**0.09**	**0.09**	**0.10**
9. CD									1.00	**0.14**	**0.14**	**0.14**
10. Opioids										1.00	**0.21**	**0.30**
11. Sedatives/tranquilizers											1.00	**0.17**
12. Stimulants												1.00

*ADHD is attention-deficit hyperactivity disorder; CD is conduct disorder. Bold correlations are significant at p < 0.05*.

### Hypothesis Tests

**Figure 1:**
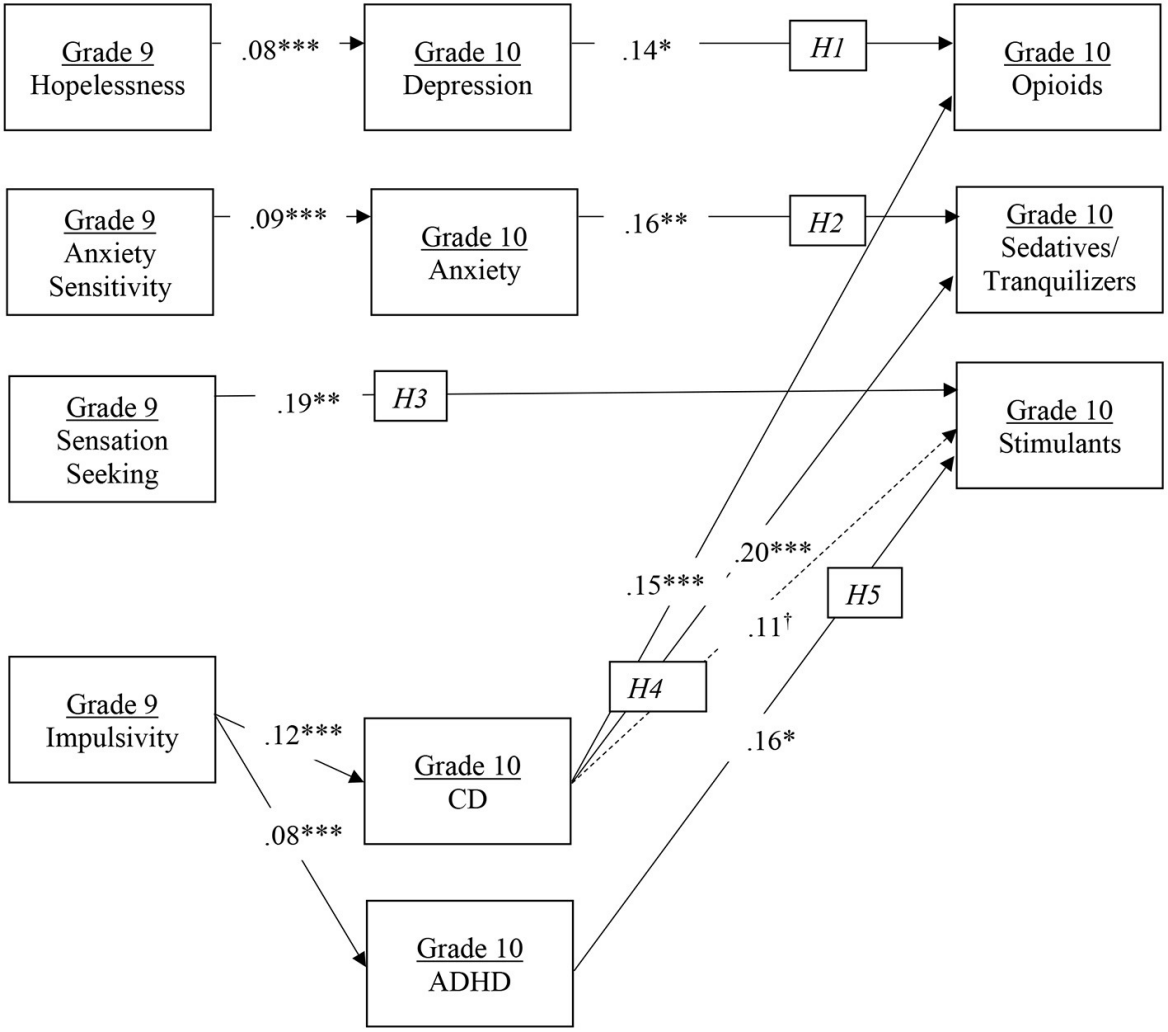
Model results. *H1-H5* represent numbered hypotheses. Solid arrows represent statistically significant hypothesized pathways; dotted arrows represent hypothesized but non-significant pathways. Numbers represent standardized coefficients. *p < 0.05, **p < 0.01, ***p < 0.001. ^†^represents marginal significance at *p* = 0.06.

Our hypothesized model (see Figure 1) showed good fit across fit indices: χ^2^(71) = 158.07, *p* < 0.001; χ^2^/*df* = 2.23; RMSEA =0.02, 90% CI [0.02, 0.03]; CFI =0.98; TLI =0.96. Indirect effects are reported in Table 4.

**Table 4:** Tests of hypothesized indirect effects.

**Hypothesis**	**Predictor**	**Mediator**	**Outcome**	**Indirect effect**	**95% confidence interval**
*H1*	Hopelessness	Depression	Opioids	0.003	[0.000, 0.007]*
*H2*	Anxiety sensitivity	Anxiety	Sedatives/tranquilizers	0.005	[0.002, 0.012]**
*H4*	Impulsivity	CD	Opioids	0.005	[0.001, 0.010]*
	Impulsivity	CD	Sedatives/tranquilizers	0.008	[0.004, 0.014]**
	Impulsivity	CD	Stimulants	0.005	[0.000, 0.014]*
*H5*	Impulsivity	ADHD	Stimulants	0.005	[0.000, 0.012]*

ADHD is attention-deficit hyperactivity disorder; CD is conduct disorder. *p < 0.05, **p < 0.01.

Grade 9 HOP significantly predicted Grade 10 depressive symptoms which in turn were significantly associated with Grade 10 opioid misuse. Consistent with *H1*, the indirect effect was statistically significant (*p* < 0.05). Grade 9 AS significantly predicted Grade 10 anxiety symptoms which in turn were significantly associated with Grade 10 sedative/tranquilizer misuse. Consistent with *H2*, the indirect effect was statistically significant (*p* < 0.01).

Consistent with *H3*, the direct path from Grade 9 SS to Grade 10 stimulant misuse was statistically significant. Grade 9 IMP significantly predicted Grade 10 CD symptoms which in turn were significantly associated with Grade 10 opioid and sedative/tranquilizer misuse and marginally associated with Grade 10 stimulant misuse (*p* = 0.06). Consistent with *H4*, all three indirect effects were statistically significant (*p* < 0.05 for opioid and stimulant misuse; *p* < 0.01 for sedative/tranquilizer misuse). Grade 9 IMP also significantly predicted Grade 10 ADHD symptoms which were in turn associated with Grade 10 stimulant misuse. Consistent with *H5*, the indirect effect was statistically significant (*p* < 0.05).

### Tests of Pathway Specificity

To determine the specificity of the HOP to opioid misuse pathway via depression symptoms [H1] and the AS to sedative/tranquilizer misuse pathway via anxiety symptoms [H2], we examined modification indices (MIs). These indicated that the inclusion of paths from AS to depression (MI: 0.23) and HOP to anxiety (MI: 2.47) did not improve model fit (values > 3.84 indicate that the model would be improved). Thus, for the sake of model parsimony, these were not added to the model.

## Discussion

### Main Findings

In the present study, we sought to address the limitations of the extant literature on adolescent PD misuse, as outlined by Nargiso et al. (20). We applied the four-factor personality vulnerability model (25) to understanding risk for misuse of specific classes of PDs in adolescents. Moreover, we applied different theoretical models of addiction (39) to understanding specific pathways from personality to adolescents' future PD misuse, as mediated through specific sets of mental health symptoms.

Different personality traits showed effects on specific types of PD misuse through unique sets of mental health symptoms, consistent with different theoretical models of addiction, namely the negative and positive affective regulation, deviance proneness, and psychological dysregulation models. Two internalizing personality traits (HOP and AS) followed a negative affect regulation model for predicting specific PD misuse, while SS (an externalizing trait) followed a positive affect regulation model. First, depressive symptoms mediated the relationship between HOP and future opiate misuse. Second, anxiety symptoms mediated the relationship between AS and future tranquilizer misuse. While both these paths are consistent with negative affect regulation, they suggest that high HOP adolescents may be using opiates to self-medicate their depressive symptoms—while high AS teens may be using tranquilizers to self-medicate their anxiety symptoms. Third, SS was predictive of future stimulant misuse suggesting high SS adolescents may be using stimulants to enhance positive affect. This suggests that adolescents high in HOP and AS are prone to PD misuse via negative affect regulation pathways while those high in SS are prone to PD misuse via a positive affect regulation pathway. Fourth, CD symptoms mediated the relationship between IMP and future opiate, sedative/tranquilizer, and stimulant misuse, consistent with a deviance proneness pathway. Unlike the other three traits, IMP therefore seems to be a more general predictor of PD misuse, rather than a specific predictor of a particular form of PD misuse. Higher IMP adolescents appear to more prone to misusing PDs indiscriminately—in the same way that they are prone to engaging in broadband antisocial behaviors. Finally, ADHD symptoms also mediated IMP's effect in the case of future stimulant misuse. We have suggested that this unique personality-to-PD misuse pathway may represent self-medication of psychological dysregulation. In the next section, we look at each of these main findings in relation to the extant literature.

### Comparison With the Literature

*H1* predicted that HOP would specifically predict future opioid misuse via depressive symptoms. This hypothesis, informed by the negative affect regulation model, was supported through a significant indirect effect from Grade 9 HOP to Grade 10 opioid misuse 1 year later as mediated through Grade 10 depressive symptoms. Depression has been identified as the mental health issue most strongly related to opioid misuse (odds ratios from 1.2 to 4.3) (87). Zullig and Divin (88) found that students who endorsed HOP, depression, and suicidality were 1.18–1.43 times more likely to misuse opioids. Opioids possess psychic pain-numbing properties (89), which may make them particularly attractive to high HOP adolescents—who are prone to depression and may be looking to dull their psychological pain. Our mediational findings are consistent with a mechanism where HOP confers risk for opioid misuse in adolescence via negative affect regulation. More specifically, high HOP adolescents may be self-medicating their depressive symptoms by misusing opioids. Given that opioids are prescribed for the management of physical pain (89) but not for the management of depression (90), any use of opioids for depression self-medication would be considered opioid misuse since it would involve taking the medication for a non-prescribed purpose (91). To help establish the specificity of this HOP risk pathway to opioid use, we tested an additional personality to PD misuse pathway informed by the negative affect regulation model involving AS (i.e., *H2*).

*H2* predicted that AS would specifically predict future sedative/tranquilizer misuse via anxiety symptoms. This hypothesis, also informed by the negative affect regulation model, was supported through a significant indirect effect from Grade 9 AS to Grade 10 sedative/tranquilizer misuse 1 year later as mediated through Grade 10 anxiety symptoms. While sedatives/tranquilizers are commonly prescribed for anxiety (92), the relevant DEP-ADO items (69) specify use “without a prescription,” suggesting that high AS adolescents may be taking non-prescribed sedatives/tranquilizers that they have obtained from family, friends, dealers, or online pharmacies (15) to self-medicate their anxiety symptoms. Taken together, support for *H1-2* suggests that there are two distinct negative affect regulation paths from personality to PD misuse. The first is specific to opioid misuse through HOP and the self-medication of depression, and the second specific to sedative/tranquilizer misuse through AS and the self-medication of anxiety. Furthermore, modification indices indicated that the inclusion of paths from AS-to-depression and HOP-to-anxiety did not improve model fit, providing further evidence of the specificity of these pathways.

Informed by the positive affect regulation model, *H3* predicted that SS would lead to future stimulant misuse. This hypothesis was supported through a direct path from Grade 9 SS to Grade 10 stimulant misuse. SS is strongly related to sensitivity to positive reinforcement and enhancement motives (31). It predicts substance misuse (93) that is driven by a need for positive affect and psycho-stimulation (29). Previously, we found that SS predicted undergraduate stimulant misuse (45). Other studies also support a robust association between SS and adolescent alcohol misuse (74). Finn et al. (94) found that SS was both directly linked to alcohol problems as well as indirectly linked through alcohol use and positive alcohol expectancies. Castellanos-Ryan et al. (95) concluded that SS's effect on binge drinking was mediated by a reward response bias. Thus, SS likely confers risk for adolescent stimulant misuse as well as excessive drinking via a positive affect regulation pathway. Taken together, the support for *H1-H3* suggests that three distinct affect regulation paths predict PD misuse in adolescence: two involving negative affect regulation (i.e., HOP to depression to opioid misuse and AS to anxiety to sedative/tranquilizer misuse) and one involving positive affect regulation (i.e., SS to stimulant misuse).

Unlike the specific associations of each of HOP, AS and SS with particular forms of PD misuse, we expected IMP to have a more general association with PD misuse, including future opioid, sedative/tranquilizer, and stimulant misuse. *H4* predicted that Grade 9 IMP would be associated with all three forms of PD misuse in Grade 10 via Grade 10 CD symptoms. These hypotheses, informed by the deviance proneness model, and IMP's centrality as a characteristic of CD (59, 96), were all supported in tests of indirect effects. This pattern is in keeping with previous research with other substances. Mackie et al. (93), for instance, found that IMP predicted adolescent alcohol use via CD symptoms. This result also replicates and extends prior research linking CD symptoms to unconstrained PD misuse in adolescents, including misuse of opioids (97) and stimulants (64). IMP's relationship with substance misuse is motivationally undefined (31) in that it is more reflective of a general inability to inhibit behavior (98). IMP is associated with deficits in response execution and inhibition (95). Poor response inhibition is a risk factor for both CD (99) and substance misuse (100). Paths from IMP to both CD and alcohol problems are also partially mediated by deficient response inhibition (94, 95). In sum, we know that high IMP adolescents struggle to regulate and inhibit their impulses. This makes them more vulnerable to deviance (including CD and PD misuse). Our results are consistent with the idea that IMP confers risk for broadband PD misuse (including all three types of PD misuse) via a general proneness toward deviance in adolescence.

In addition to these general IMP to CD symptoms to PD misuse pathways, *H5* predicted a second indirect pathway specifically linking IMP to later stimulant misuse via ADHD symptoms. This hypothesis, informed by the psychological dysregulation model, was supported by a significant indirect effect from Grade 9 IMP to Grade 10 stimulant misuse via Grade 10 ADHD symptoms. IMP is a prominent symptom of ADHD (101) for which stimulants are prescribed (102). Previously, we showed that IMP was concurrently associated with both medically sanctioned stimulant use and stimulant misuse in university students (45). Prescription stimulants are classified as Schedule III under the Canadian Controlled Drugs and Substances Act (S.C. 1996, C. 19) due to their high potential for misuse (103). Their use is legal only when prescribed by a licensed practitioner and taken by the person for whom they were prescribed. For those high in IMP, availability is the best motivational predictor of misuse (34). Adolescents who report symptoms of ADHD are more likely to have stimulant prescriptions, which they can then misuse [e.g., by taking their stimulants in greater amounts or more often than prescribed, via non-intended routes, for non-prescribed reasons, and/or with contraindicated substances; (91)]. While rates of stimulant misuse are relatively low in general adolescent samples, rates are much higher among adolescents who: have symptoms of ADHD, have ADHD diagnoses, are receiving treatment for ADHD, or have stimulant prescriptions (104). Interestingly, some research suggests that the young people most likely to misuse prescription stimulants are those with markers of a possible mental health disorder (e.g., ADHD) but without a formal diagnosis or prescription (105). Our results suggest that some young people may misuse stimulants to cope with their ADHD-related disorganization, poor time management, forgetfulness, and distractibility (64). Thus, in adolescence, IMP may confer risk for stimulant misuse, in part, via self-medication of psychological dysregulation—a form of self-medication that is theoretically distinct from the self-medication of negative affect pathways described above for AS and HOP.

### Strengths and Limitations

Our study has several important strengths. These include the large sample size, inclusion of both French- and English-speaking students, the longitudinal component (personality to mental health symptoms and personality to PD misuse paths) over a 1-year follow-up across the developmentally challenging transition to high school, the excellent retention rate (95%), the control of baseline levels of mediators and outcomes in all models, and the theoretically driven hypotheses. Moreover, the topic of the paper is likely to be of interest to both a general and specialty audience of mental health professionals, particularly those that work with youth.

These findings should be interpreted in the context of several potential study limitations. First, we measured personality in Grade 9—and mental health symptoms *and* PD misuse in Grade 10. As such, the final pathways in our semi-longitudinal model (from mental health symptoms to PD misuse) were cross-sectional. Methodologically, we set up our semi-longitudinal model in this manner because *H1-H3* pertain to self-medication. We considered assessing PD misuse in Grade 11, in a three-wave design, but this would have meant testing whether students misused PDs to cope with the mental health symptoms they had reported a year earlier. We wanted to measure mental health symptoms and PD misuse in closer proximity. Self-medication models posit that the mental health-to-PD misuse relationship is unidirectional (50). There are data, however, that suggest that it may be bidirectional. PD misuse, for example, has been shown to exacerbate students' mental health symptoms (106). Our data do not allow us to compare these possibilities and our model does not allow for causal inference. Nonetheless, mediation analyses with even partially cross-sectional data can be a useful starting point (107) and our model had the advantage of being semi-longitudinal (i.e., where part of the design was longitudinal—specifically personality to mental health symptoms and personality to PD misuse). To demonstrate reliability and address these limitations, however, our model should be replicated in a fully longitudinal design that uses shorter (e.g., 6 month) lags between waves. Future research could also use ecological momentary assessment to examine these relationships day-to-day [e.g., (108)].

A second potential limitation pertains to our measure of PD misuse. The DEP-ADO was chosen because it is standardized, has been demonstrated reliable and valid in the measurement of Canadian high school students' substance use (69), and can be use with both English- and French-speaking Québécois adolescents (73). Despite these strengths, the DEP-ADO has some shortcomings. For example, we assessed each type of PD misuse with a single item, introducing measurement error. It also provides little information about students' means of access (e.g., diversion sources, online pharmacies), administration routes, or motives for use. Moreover, different definitions of PD misuse abound (109), and it has been suggested that none of the instruments published to date can adequately assess PD misuse (110). When improved PD misuse measurement tools become available, our model should be replicated. This would reduce measurement error, allowing for a more accurate and refined test of personality's effects on PD misuse generally and on specific classes of PD misuse specifically.

Third, our sampling was limited. While our study was bolstered by its large sample size, this increases the likelihood that small effects will be statistically significant. And some of our effects were relatively small in magnitude, calling for evaluation of their clinical significance (see below). In addition, the students who did not complete our Grade 10 measures were more likely to report Grade 9 personality vulnerability, mental health symptoms, and alcohol and PD misuse, and were more likely to come from specific schools. Some of these results are in keeping with previous studies, in which adolescents lost to follow up were more likely to be involved in drug use and other deviant behavior (111–113). Moreover, we controlled effects of school in our analyses. It still bears noting, however, as samples and findings can be biased when the individuals who drop out differ substantially from those who are retained (114).

Finally, while the use of our brief personality measure [SURPS; (31)] allowed for brevity in the context of a large-scale survey, it did not allow for nuanced assessment of the components of each of our traits. For example, the longer Childhood Anxiety Sensitivity Index (115) would have allowed for examination of the relative contributions of the AS Physical, Social/Control, and Psychological concerns dimensions (116) to the anxiety symptom mediated pathway to sedative/tranquilizer misuse observed in the present study. Similarly, the longer Barratt Impulsiveness Scale (117) would have allowed for examination of the relative contributions of the Attentional, Motor, and Non-planning Impulsiveness components (118) to the CD and ADHD symptom mediated pathways to PD misuse observed in the present study.

### Future Research Directions

The present study focused on the mediating effects of mental health symptoms. Motives for PD misuse were not assessed. Bennett and Holloway (119) have concluded that opioids, sedatives/tranquilizers, and stimulants tend to be misused in one of two ways. PDs are misused for self-medication of mental health (e.g., more sleep, less anxiety) or physical health (e.g., to manage a pre-existing illness) problems. They are also misused for pleasure (e.g., to party, get high, or experiment). Boyd et al. (22) and McCabe et al. (46) have published measures of motives for PD misuse. Negatively and positively reinforcing motives are both associated with increased PD misuse frequency (120). Follow-up studies might test whether personality predicts specific motives for PD misuse just as personality predicts specific motives for alcohol use (121). Previously, in the alcohol field, we found chained mediation from personality to mental health symptoms, to drinking motives, to alcohol outcomes (122). The results of the present study suggest that a four-variable chained mediational model might be equally applicable to PD misuse. For example, HOP may predict opioid misuse via symptoms of depression and in turn self-medication motives.

There are also several other areas of future research that are worthy of investigation in the field of personality and PD misuse risk more broadly. First, given that online marketplaces are an accessible source of PDs for young people [e.g., largely uncontrolled, not requiring a prescription, allowing for anonymous access; (123, 124)], and thus a significant public health concern, we need more information on the types of adolescents who are accessing PDs via these sites. While the demographic characteristics of the typical customers of such online marketplaces have been identified [i.e., young, male, Caucasian; (125)], we have not yet identified their personality or mental health characteristics, which would be helpful for targeting prevention efforts. Second, given the well-established role of social influence in young people's drug misuse [e.g., (126)] and emerging data concerning online drug forums and social networking sites where those experimenting with psychotropics, including PDs, share drug-related information (9), it would be interesting to study whether involvement in such communities might be related to personality. For example, are these experimenters or “psychonauts” higher in sensation seeking? Finally, personality and mental health factors may be relevant when it comes to pre-marketing assessment trials of the abuse liability of new prescription drugs. Current practices in this regard have been criticized for excluding those with a previous history of drug misuse or addiction [e.g., (8)]. Given the present findings of significant links of four factor personality model traits and mental health symptoms to different forms of PD misuse, there could be utility to testing a new compound's abuse potential using these more substance-misuse prone individuals in pre-marketing assessment trials to get at the compound's truer abuse liability.

### Clinical Implications

Our model suggests that treatment of opioid misuse in adolescents might benefit from a specific targeting of HOP and IMP youth. Cognitive-behavioral therapy (CBT) could benefit teens high in HOP, by teaching them to better cope with their symptoms of depression (127). Motivational approaches could benefit antisocial teens high in IMP, by increasing their future-oriented thinking and teaching them to weigh the short vs. long term consequences of their behavior (128). Because we substantiated paths from IMP to CD symptoms, to opioid, sedative/tranquilizer, and stimulant misuse—a focus on this personality factor would theoretically reduce misuse of a variety of types of PDs. The results of our specificity tests further suggest that treatments of sedative/tranquilizer misuse be targeted toward youth high in AS and include techniques drawn from CBT for anxiety (128). To treat stimulant misuse, our model suggests we should be targeting adolescents high in externalizing traits. Those high in SS could be encouraged to pursue other stimulating yet prosocial activities (129). “Alternate rebellions” including hair dyeing, getting a tattoo, or getting a piercing (130) are safer activities that might meet these adolescents' need for excitement. In contrast, psychologically dysregulated, high-IMP teens could be trained in behavioral ADHD-management techniques (131).

Treating PD misuse is, of course, important. But, given the ongoing PD crisis in North America (132), preventing it is *critical*. Adolescent overdoses from prescription opioids rose 95% from 1999 to 2016 (133). The likelihood of reporting PD misuse during adolescence, increases with age (83), as we saw across each PD type from Grade 9 to 10 in our sample. Research has shown that PD misuse rates rise consistently between Grade 8–12 and ages 12–17 (134). Thus, prevention efforts geared toward at-risk youth are especially vital. Our results suggest that identifying high personality-risk adolescents (i.e., those high in HOP, AS, SS, or IMP) would benefit both early intervention and targeted prevention strategies for PD misuse.

Personality-matched interventions have effectively reduced illicit drug use in adolescence (135) and PD misuse in adulthood (136). The present study was embedded within a larger trial, which evaluated the longer-term efficacy of the Preventure Program (65). This personality-matched prevention program targets teens with elevated four-factor trait scores (25). It is rooted in the cognitive-behavioral model and incorporates psycho-educational and motivational interviewing components. When applied to alcohol and illicit drug use, the Preventure Program has resulted in delayed onset and reduced escalation of misuse (65). Our study suggests that personality is related to PD misuse in a similar manner to its relations with alcohol and illicit drug use, through mental health symptoms. Thus, personality-matched interventions may have the potential to reduce PD misuse and even prevent PD uptake, if administered prior to PD misuse onset. Our results suggest that the Preventure Program should next be investigated in relation to its utility in targeting adolescent PD misuse.

## Data Availability Statement

The raw data supporting the conclusions of this article will be made available by the authors, without undue reservation.

## Ethics Statement

Ethical approval was granted by Sainte-Justine Hospital's Research Ethics Board and each administrative school board. The consent process varied across schools. Some schools opted for active consent where written informed consent to participate in the study was provided by the participants' legal guardian/next of kin. Other schools opted for a passive consent procedure where legal guardians/next of kin were fully informed about the study and they declined if they did not consent for their adolescent to participate. All adolescents provided their assent prior to participating.

## Author Contributions

SS and AC wrote the manuscript with input from all co-authors. KT ran the statistical analyses. MA assisted with database management. The data collection was coordinated by PC as part of the CoVenture trial. All authors assisted with conceptualization of the model and interpretation of the results.

## Conflict of Interest

The authors declare that the research was conducted in the absence of any commercial or financial relationships that could be construed as a potential conflict of interest.
